# High-density lipoprotein inhibits mechanical stress-induced cardiomyocyte autophagy and cardiac hypertrophy through angiotensin II type 1 receptor-mediated PI3K/Akt pathway

**DOI:** 10.1111/jcmm.12567

**Published:** 2015-05-06

**Authors:** Li Lin, Xuebo Liu, Jianfeng Xu, Liqing Weng, Jun Ren, Junbo Ge, Yunzeng Zou

**Affiliations:** a Department of Cardiovascular Medicine, East Hospital, Tongji University School of MedicineShanghai, China; b Shanghai Institute of Cardiovascular Diseases, Zhongshan Hospital and Institute of Biomedical Science, Fudan UniversityShanghai, China

**Keywords:** autophagy, cardiac hypertrophy, heart failure, AT1 receptor, HDL

## Abstract

Mechanical stress triggers cardiac hypertrophy and autophagy through an angiotensin II (Ang II) type 1 (AT1) receptor-dependent mechanism. Low level of high density lipoprotein (HDL) is an independent risk factor for cardiac hypertrophy. This study was designed to evaluate the effect of HDL on mechanical stress-induced cardiac hypertrophy and autophagy. A 48-hr mechanical stretch and a 4-week transverse aortic constriction were employed to induce cardiomyocyte hypertrophy *in vitro* and *in vivo*, respectively, prior to the assessment of myocardial autophagy using LC3b-II and beclin-1. Our results indicated that HDL significantly reduced mechanical stretch-induced rise in autophagy as demonstrated by LC3b-II and beclin-1. In addition, mechanical stress up-regulated AT1 receptor expression in both cultured cardiomyocytes and in mouse hearts, whereas HDL significantly suppressed the AT1 receptor. Furthermore, the role of Akt phosphorylation in HDL-mediated action was assessed using MK-2206, a selective inhibitor for Akt phosphorylation. Our data further revealed that MK-2206 mitigated HDL-induced beneficial responses on cardiac remodelling and autophagy. Taken together, our data revealed that HDL inhibited mechanical stress-induced cardiac hypertrophy and autophagy through downregulation of AT1 receptor, and HDL ameliorated cardiac hypertrophy and autophagy *via* Akt-dependent mechanism.

## Introduction

Autophagy is a highly conserved cellular process for protein degradation and recycling. Initially described as a cell survival mechanism in starvation, autophagy has recently gained much attention as an avenue for cell death in a number of diseases including heart failure 1. Excessive autophagy was found in patients with heart failure 2,3 and is believed to contribute to the onset and progression of cardiac remodelling and myocardial damage elicited by mechanical stress 4–6. However, the few available autophagy inhibitors cannot be used in patients because of toxicity issues thus making it pertinent to search for other routes for inhibition of autophagy.

High density lipoprotein (HDL) exhibits antiatherogenic and cardioprotective effects 7,8. In addition to its classical action in the reverse cholesterol transport, HDL exerts antioxidant and anti-inflammatory effects in patients with heart failure 9,10. The Framingham Heart Study demonstrated an enduring relation between low HDL cholesterol and incidence of heart failure after exclusion of baseline coronary heart diseases 11. It has also been reported that HDL is more superior to B-type natriuretic peptide as a marker of systolic cardiac dysfunction in the elderly 12. Moreover, low levels of HDL cholesterol may predict unfavourable prognosis in patients with heart failure, independent of the nature of aetiology 13. Direct cellular effects of HDL have been demonstrated in cardiomyocytes 14. Furthermore, our previous study exhibited that HDL is capable of inhibiting angiotensin II (Ang II)-induced cardiac hypertrophy 15. More recently, HDL was found to suppress autophagy 16,17.These data collectively suggest that HDL may play a unique role in the suppression of autophagy and cardiac hypertrophy. Nonetheless, the precise role of HDL in pressure overload-induced autophagy and cardiac hypertrophy remains elusive.

## Materials and methods

### Animal models

All animal procedures described here were approved by the Animal Care and Use Committee of Fudan University and were in compliance with the Guidelines for the Care and Use of Laboratory Animals published by the National Academy Press (NIH Publication No. 85-23, revised in 1996). In brief, adult male C57BL/6 mice (aged 8–10 weeks) were obtained from the Jackson Laboratory (Bar Harbor, ME, USA) and were subjected to transverse aortic constriction (TAC) or sham operation under anaesthesia (ketamine, 25 mg/kg, i.p.) as described 18. Following anaesthesia and artificial ventilation, transverse aorta was constricted with a 7-0 nylon suture by ligating the aorta using a blunted 27-gauge needle. The needle was extracted immediately after the procedure. HDL particles were purchased from Calbiochem which are from human sources. 100 μl was used for HDL administration. HDL (400 ng/kg/min) or PBS (used as a vehicle) was continuously administered using Alzet osmotic minipumps (Model 2002; DURECT, Cupertino, CA, USA) implanted subcutaneously into the back of mice 3 days prior to TAC. MK-2206 (100 mg/kg, 3 times per week) was administered *via* a stomach tube 3 days prior to TAC procedure until 4 weeks after. Four weeks following the TAC procedure, all mice were killed and hearts were excised for further examination.

### Echocardiography and hemodynamic measurements

Transthoracic echocardiography was performed with a 30-MHz high-frequency scan head (Visual Sonics Vevo770; Visual Sonics Inc., Toronto, ON, Canada) 19. All measurements were carried out by three experienced technicians who were blinded to experimental group identity. Five consecutive cardiac cycles were used for average. Blood pressure (BP) was evaluated as described 20–22. A micromanometer catheter (Millar 1.4F, SPR 835; Millar Instruments, Inc., Houston, TX, USA) was inserted into the right common carotid artery and the transducer was connected to a Power Laboratory system (AD Instruments, Castle Hill, NSW, Australia) for BP recording.

### Morphology and histological analyses

Excised hearts were weighed, perfused with PBS, and fixed with 4% polyformaldehyde for global morphometry and then with 10% formalin for histological analysis. The papillary muscle levels were the hearts excised for LV characterization of cardiomyocyte hypertrophy. We took serial cuts to evaluate cardiomyocyte hypertrophy. Paraffin-embedded hearts were sectioned at a thickness of 4 μm and stained with hematoxylin and eosin. Cardiomyocyte morphology and histology were examined under high magnification to assess cross-sectional area (CSA) using a video camera (Leica Qwin 3, Leica Microsystems, Wetzlar, Germany) attached to a micrometre. Twenty randomly chosen fields were evaluated from each cross section of the LV free wall.

### Cell culture and treatment

Neonatal cardiomyocytes were prepared from 1 to 3 days Sprague–Dawley rats by trypsin digestion method as described elsewhere. Briefly, neonatal rat ventricles were minced into pieces and subjected to 0.125% trypsin digestion in hank’s balanced salt solution. After 2 hrs of cell attachment, cardiomyocytes were collected and maintained for 48 hrs in DMEM/F12 containing 10% FBS with antibiotics. Then serum was deprived for 24 hrs prior to experiments. Isolated neonatal cardiomyocytes were cultured in the silicon-based culture dishes pre-coated with collagen. Mechanical stress stimulation was induced by stretching the silicon culture dishes by 120% in the specifically produced stretch equipment 18. HDL (100 μg/ml) or MK-2206(100 nM) was pre-administered in the culture medium for 30 min. The mechanical stretch was applied for 48 hrs, and cardiomyocytes were collected to extract protein and total RNA for further study. MK-2206, a selective inhibitor for Akt phosphorylation, was used to determine the relative contribution of Akt phosphorylation to the action of HDL.

### [^3^H] leucine incorporation

Cultured cardiomyocytes were incubated with [^3^H] leucine (1 μCi/ml) in silicon-based plates pre-coated with collagen. The cells were then subjected to a 48-hr mechanical stretch before exposure to 5% trichloroacetic acid. Protein precipitates were dissolved in 1 ml of 100 mmol/l NaOH, and radioactivity was determined using a liquid scintillation counter.

### Real-time RT-PCR

Total RNA was isolated from LV tissues or cultured cells using TRIzol® reagent according to the manufacturer’s instructions. After purification, RNA was subjected to real-time RT-PCR analysis to determine the expression of *atrial natriuretic peptide* (*ANP*) and *skeletal*α*-actin* (*SAA*) using a BIO-RAD IQ5 multicolor detection system (Hercules,CA,USA). The melting curves and quantitation were analysed using the Light Cycler and Rel Quant software programs, respectively. The comparative CT method was used to determine relative quantification of RNA expression 23. All PCR reactions were performed at least in triplicate.

### Western blot analyses

The homogenized ventricles tissue or cultured cells were incubated in lysis buffer (pH 7.4) containing 50 mM Tris-HCl (pH 7.4), 150 mM NaCl, 5 mM EDTA, 25 mM NaF, 1% Triton-X 100, 1% NP-40, 0.1 mM Na_3_VO_4_, 12.5 mM b-glycerophosphate, 1 mM phenylmethanesulfonylfluoride (PMSF) and complete protease inhibitor cocktail. Insoluble heart tissues or cell fractions were removed by centrifugation at 13,000 × g, 4°C for 30 min. The concentration of the extracted proteins was measured by bicinchonininc acid assay (Sigma-Aldrich, St. Louis, MO, USA). The extracted proteins were boiled for 3–5 min. in 5× loading buffer. The expression of AT1 receptor, LC3b-II, beclin-1 and phosphorylation of Akt (*p*-Akt) were detected by immune-blotting with antibodies against AT1 receptor (catalogue no.sc-1173G; Santa Cruz Biotechnology, Santa Cruz, CA, USA), LC3b-II (catalogue no. 2775; Cell Signaling Technology, Danvers, MA, USA), beclin-1 (catalogue no. 3495; Cell Signaling Technology), and *p*-Akt (catalogue no. 9275; Cell Signaling Technology), and detection was performed by using an ECL Western-blotting Detection Reagents (RPN2106; GE Healthcare, Little Chalfont, Buckinghamshire, UK) visualized densitometry with LAS-300 Image software.

### Immunofluorescence

After incubation for 48 hrs on silicon-based plates in serum-free DMEM medium, cardiomyocytes were incubated with the anti-α-MHC (catalogue no. 05-716; Upstate, Millipore Corporation, Billerica, MA, USA) and anti-LC3b-II (catalogue no. 2775; Cell Signaling Technology) antibodies. The samples were then incubated with secondary antibodies conjugated to FITC or Alexa (catalogue no. A21206; Invitrogen, Carlsbad, CA, USA) according to the manufacturer’s instructions. The surface area (SA) of cardiomyocytes and LC3b-II staining were determined using image analysis software (Leica Qwin3) and were calculated using the mean of 100 to 120 cells from randomly selected fields.

### Terminal dUTP nick end-labelling and immunohistochemical staining double-labelling assay

A double-labelling assay for detecting apoptotic cells, using TUNEL assay and antigens of LC3b-II was performed. Briefly, cardiomyocytes were incubated with anti-LC3b-II antibody. The samples were then incubated with secondary antibodies according to the manufacturer’s instructions.Apoptotic death of cardiomyocytes was detected *in situ* by terminal deoxyribonucleotidetransferase-mediated dUTP nick-end labelling (TUNEL) kit (TaKaRaBiomedicals, Otsu, Shiga, Japan) in cardiomyocytes according to the manufacturer’s direction. The LC3b-II positive cardiomyocytes and TUNEL positive cells were stained. Immunoreactive cells in 20 fields from each sample by using the 40× objective and TUNEL-positive nuclei in the same fields by using the same power objective.

### Statistical analysis

Data were shown as means ± SEM. Comparisons were performed by one-way anova followed by the Newman–Keuls test for *post-hoc* analysis to determine the differences among the groups.

## Results

### HDL inhibited mechanical stress-induced hypertrophic and autophagic responses in cultured cardiomyocytes

**Figure 1:**
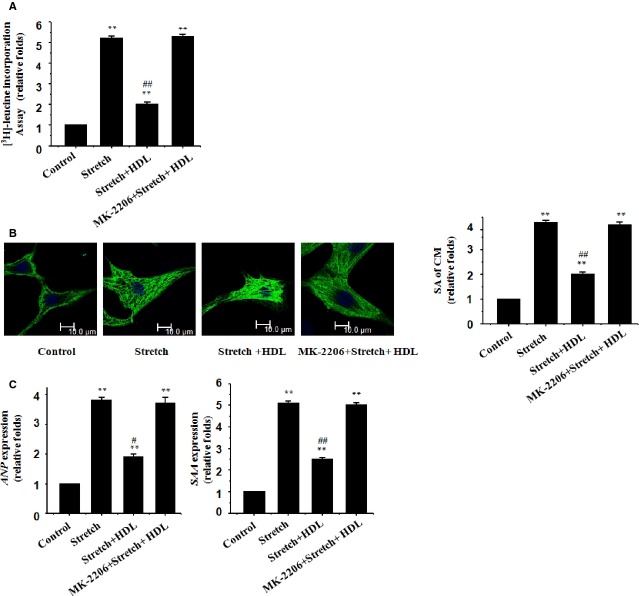
HDL inhibits mechanical stress-induced hypertrophic responses in cultured cardiomyocytes. Cultured rat neonatal cardiomyocytes were treated with vehicle (control) or mechanical stretch in the absence or presence of HDL (100 μg/ml), MK-2206 (100 nM) or both; (A) [^3^H]-Leucine incorporation in cardiomyocytes; mean ± SEM from 3 independent assays. (B) Cardiomyocyte morphology and size in cardiomyocytes subjected to immunofluorescence staining for α-MHC (green) and DAPI. Representative photographs from 3 independent experiments are shown (scale bar: 10 μm). The surface area (SA) of cardiomyocytes was evaluated by measuring 100 cardiomyocytes in each dish. (C) Expression of the *ANP* and *SAA* genes evaluated by real-time RT-PCR; β-actin was the internal control.

The rate of protein synthesis in cultured rat neonatal cardiomyocytes was examined. The results showed that mechanical stretch overtly increased protein synthesis in cardiomyocytes (Fig.1A). Mechanical stretch also significantly increased the SA of cardiomyocytes measured using immunostaining with an anti-α-MHC antibody (Fig.1B). Consistently, the levels of the foetal-type genes ANP and SAA were up-regulated in stretched cardiomyocytes (Fig.1C). Our data further revealed that administration of HDL significantly suppressed mechanical stretch-induced hypertrophic responses including greater rate of protein synthesis, enlarged SA, and appearance of reprogrammed foetal gene (Fig.1A–C), indicating that an anti-hypertrophic property for HDL against mechanical stretch *in vitro*.

**Figure 2:**
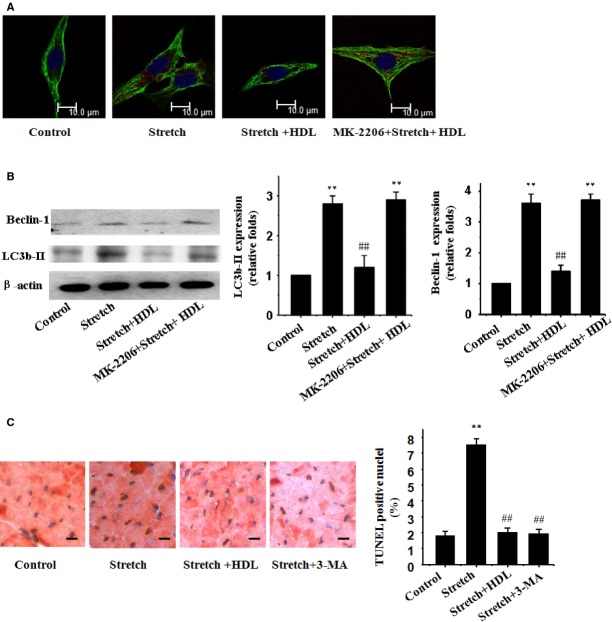
HDL inhibits mechanical stress-induced autophagy in cultured cardiomyocytes. (A) Immunofluorescence staining of LC3b-II (red) and α-MHC (green). (B) Western blot analyses for LC3b-II and beclin-1 using the anti LC3b-II and anti beclin-1 antibody; β-actin in whole cell lysate was used as the loading control. Representative photograms from 3 independent experiments are shown. (C) TUNEL and immunohistochemical staining double- labelling assay. Mean ± SEM from 3 independent experiments, **P < 0.01 *versus* cardiomyocytes in the control; ^##^*P* < 0.01 *versus* cardiomyocytes with stretch.

Our results further revealed a significantly increased cardiomyocyte autophagy as demonstrated by LC3b-II and beclin-1 in neonatal cardiomyocytes following a 48-hr mechanical stretch (Fig.2A and B), the effect of which was remarkably suppressed by HDL (Fig.2A and B).

To investigate the role of autophagy in the mechanism of apoptosis induced by mechanical stress, some experiments were performed in the presence of 3-methyladenine (3-MA), an inhibitor of the initial stages of autophagy. 3-MA prevented the increase in autophagy induced by mechanical stress and, more importantly, completely antagonized apoptosis induced by mechanical stress (Fig.2C). HDL also suppressed apoptosis induced by mechanical stress. Overall these results clearly indicate that mechanical stress induces autophagic apoptosis and the effect of which was remarkably suppressed by HDL.

### HDL inhibited mechanical stress-induced cardiac hypertrophy and autophagy *in vivo*

**Figure 3:**
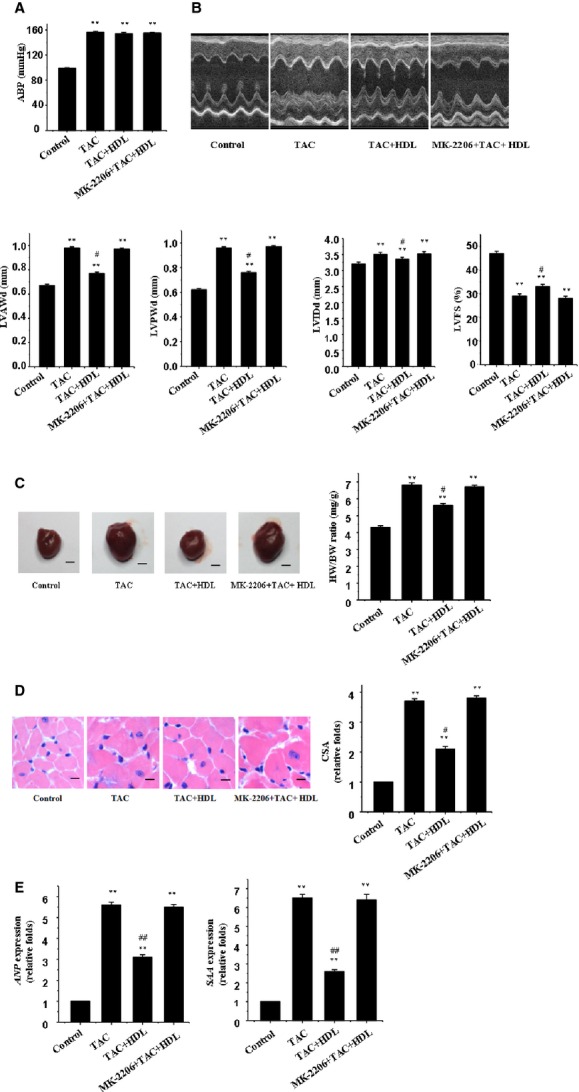
HDL inhibits mechanical stress-induced cardiac hypertrophy in mice. (A) BP recordings. Representative recordings from 5 mice are shown. (B) Echocardiographic analysis with representative M-mode tracings from 5 mice. Mean ± SEM from 5 mice. LVAWd: LV anterior wall thickness at end-diastole; LVPWd: LV posterior wall thickness at end-diastole; LVIDd: LV internal dimension at end-diastole; LVFS: LV fraction shortening. (C) Heart morphology and weight. Representative global heart photographs of 5 mice are shown (scale bar: 2 mm), and the heart weight to body weight ratio (HW/BW) was measured in 5 mice. (D) Haematoxylin and eosin stained LV sections of mice. The cross-sectional area (CSA) of cardiomyocytes measured using 5 sections from one heart and 5 hearts was examined (scale bar: 20 μm). (E) Expression of the *ANP* and *SAA* genes was evaluated by real-time RT-PCR. β-actin served as the internal control.

Four weeks following TAC, all mice were alive. Cardiac function and BP were examined. Our data revealed an approximate 55% rise in BP following the pressure overload procedure (Fig.3A). Echocardiographic evaluation showed that TAC-operated mice developed apparent cardiac hypertrophy, including signs of increased LV anterior wall during end diastole, LV posterior wall during end diastole, and LV internal dimension during end diastole, together with decreased LV fractional shortening (Fig.3B). Gross heart size and the ratio of heart-to-body weight (HW/BW) were also increased following pressure overload (Fig.3C). Analysis of cardiomyocyte SA in hematoxylin and eosin stained LV sections revealed that pressure overload significantly enlarged cardiomyocyte size (Fig.3D). Similar to the results from *in vitro* experiments, the up-regulation of SAA and ANP was also observed in the hearts from TAC-operated mice (Fig.3E).

**Figure 4:**
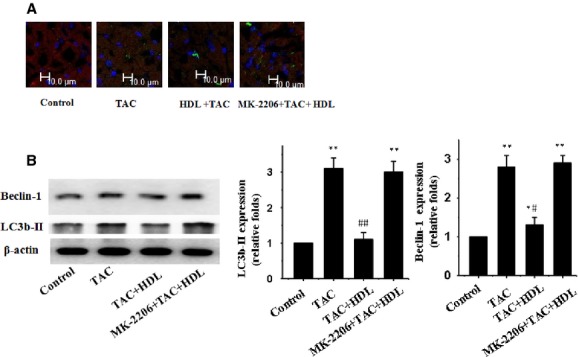
HDL inhibits mechanical stress-induced autophagy in mice. (A) Immunofluorescence staining was performed to examine the expression of LC3b-II proteins using an anti-LC3b-II antibody (green); the hearts were also subjected to immunofluorescence staining for α-MHC (red) and DAPI staining. (B) Western blot analyses for LC3b-II and beclin-1 using the anti LC3b-II and anti beclin-1 antibody; β-actin in whole cell lysate was used as the loading control. Representative photograms from 3 independent experiments are shown. Mean ± SEM of 5 mice in all groups. *P < 0.05, **P < 0.01 *versus* control, ^#^*P* < 0.05, ^##^*P* < 0.01 *versus* TAC-treated group.

Consistent with the anti-hypertrophic effect of HDL upon mechanical stretch stimulation in cultured cardiomyocytes, HDL inhibited pressure overload-induced cardiac hypertrophy, as demonstrated by the thinner ventricular wall and preserved systolic function (Fig.3B). The HW/BW ratio, SAA, ANP, and cell SA of cardiomyocytes were all remarkably inhibited by HDL (Fig.3C–E). Our result further depicted apparent autophagy in response to pressure overload, as shown by LC3b-II and beclin-1 (Fig.4A and B). Consistent with the *in vitro* finding, HDL dramatically suppressed mechanical stress-induced cardiomyocyte autophagy *in vivo* (Fig.4A and B).

### HDL downregulated AT1 receptor level in mechanical stress-treated cardiomyocytes

**Figure 5:**
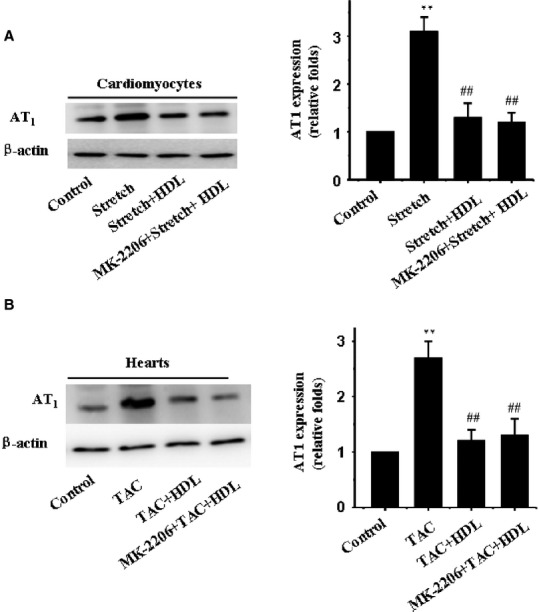
Effect of HDL on the expression of AT1 receptor. Membrane proteins were extracted from cardiomyocytes and LV of mice. Western blot analyses were employed to determine the expression of AT1 receptor using an anti-AT1 receptor antibody. β-actin was used as the loading control. Representative photograms from 3 independent experiments with cardiomyocytes or from 5 mouse hearts are shown.

The AT1 receptor is believed to play an essential role in the development of cardiac hypertrophy and autophagy 24. Here, we tested whether HDL-elicited inhibitory effect on mechanical stress-induced cardiac hypertrophy and autophagy was due to inhibition of AT1 receptor. Our data revealed that mechanical stress triggered up-regulation of AT1 receptor in both cultured cardiomyocytes and hearts, the effects of which were reconciled by HDL (Fig.5). These findings suggest that downregulation of the AT1 receptor may contribute to HDL-elicited beneficial effect against cardiac hypertrophy and autophagy.

### PI3K/Akt pathway involves in the protective effect of HDL

**Figure 6:**
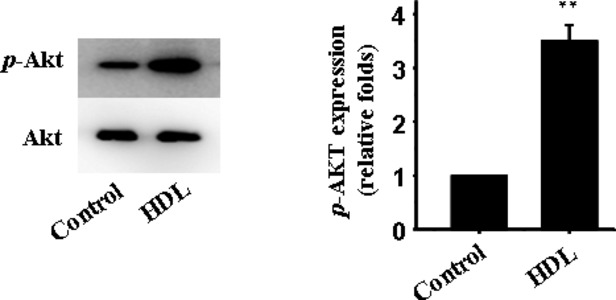
Effect of HDL on the expression of *p*-Akt. Western blot analysis for expression of *p*-Akt; Akt in whole cell lysate used as the loading control; mean ± SEM of 5 mice in all groups. **P < 0.01 *versus* control.

As previously reported, PI3K/Akt pathway played a critical role in autophagy 25. To determine if HDL exerts its action through PI3K/Akt pathway, we examined the phosphorylation levels of Akt in HDL-treated cardiomyocytes. The phosphorylation was significantly increased by HDL (Fig.6). Interestingly, MK-2206, a selective inhibitor for Akt phosphorylation, prevented HDL-elicited response against mechanical stress-induced autophagy both *in vitro* (Fig.2A and B) and *in vivo* (Fig.4A and B).

## Discussion

Earlier evidence has indicated that HDL protects against atherosclerosis through reverse cholesterol transport 26. Nonetheless, increasing reports have demonstrated that HDL exerts biological actions related to anti-inflammatory, anti-oxidative, and anti-apoptotic properties independently of reverse cholesterol transport. More recent evidence has demonstrated that HDL inhibits autophagy 17. These biological functions may contribute to cardiac events associated with HDL 27–31. Findings from our current study revealed that HDL overtly inhibited the development of cardiac hypertrophy, dysfunction, and autophagy following 4 weeks of pressure overload in mice, suggesting that a beneficial role for HDL in preserving cardiac homeostasis during pressure overload. In addition, these data suggest that autophagy regulation may be responsible for HDL-induced beneficial effects in the myocardium, possibly through inhibition of AT1 receptor *via* a PI3K/Akt-dependent mechanism.

Mechanical stress is the most important stimulus that triggers hypertrophic responses. Our present study demonstrated a protective property for HDL against mechanical stress-induced cardiomyocyte hypertrophy. As manifested by the heart size (HW/BW ratio), cardiomyocyte area, echocardiographic properties, and mRNA level of ANP and SAA, cardiac hypertrophy was clearly induced by a 4-week period of mechanical stress. HDL significantly alleviated mechanical stress-induced cardiac hypertrophic responses. These findings indicated that HDL is capable of counteracting mechanical stress-induced cardiac hypertrophy.

Autophagy, an evolutionarily conserved process of lysosome-dependent turnover of aged and damaged proteins and organelles 32, plays a pivotal role in the maintenance of cardiac homeostasis 5,33. At basal level, autophagy performs housekeeping function to maintain cardiac function and ventricular mass. However, autophagy may become excessive in the face of cardiac hypertrophy and may play a role in the transition from cardiac hypertrophy to heart failure 24,34. Autophagy has gained much attention as a main route of cell death in heart failure 1,35. Recent studies reported that HDL inhibited autophagy in endothelial cells and adipocyte 16,17. However, the precise role of HDL in pressure overload-induced autophagy remains unknown. Data from our studies revealed that HDL inhibited mechanical stress-induced autophagy both *in vitro* and *in vivo*. These results favour a unique role for HDL in mechanical stretch-induced cardiomyocyte autophagy. We also examined the apoptotic response following autophagy. The role of autophagy and apoptosis in mechanical stress-induced cardiomyocyte death was detected by TUNEL method, in the presence of 3-MA. 3-MA prevented the increase in not only autophagy but also apoptosis induced by mechanical stress. Treatment with HDL also suppressed both the autophagic and apoptotic cell death induced by mechanical stress, which is consistent with the effect of 3-MA. It has been indicated that pressure overload-induced cardiac hypertrophy is initiated by a wave of apoptosis of cardiomyocytes which is involved in the pathogenesis of cardiac remodelling. Collectively, since HDL can not only decrease excessive autophagy but also inhibit apoptotic cardiomyocyte death induced by mechanical stress, it therefore improves cardiac hypertrophy and heart function.

It is widely known that the AT1 receptor plays a critical role in the development of mechanical stress-induced cardiac hypertrophy 36–38, and cardiac dysfunction 39–41. Accumulating evidence has recently indicated that overactivation of AT1 receptor may be associated with autophagy induction 42–44. Porrello and colleagues first revealed a possible interplay between Ang II and autophagy in neonatal cardiomyocytes 45. Subsequent work showed that Ang II promoted autophagic activity in podocytes, leading to renal injury 46. Our previous study has shown that mechanical stress triggered cardiomyocyte autophagy through AT_1_ receptor 47. Given that AT1 receptor is activated in cardiac hypertrophy and autophagy, HDL has been suggested to regulate AT1 receptor expression during cardiac hypertrophy and autophagy. To test this hypothesis, mice were administered HDL *in vivo* whereas cardiomyocytes were exposed to mechanical stress prior to exposure to HDL *in vitro*. HDL-induced downregulation of AT1 receptor suggested an antagonistic property for HDL against mechanical stress-mediated cardiac hypertrophy and autophagy. In our hands, the HDL-induced downregulation of AT1 receptor in cardiomyocytes was consistent with a decrease in cardiac hypertrophy and autophagy. These results suggested that the downregulation of AT1 receptor by HDL may serve as an underlying mechanism for the subsequent reduction in cardiac hypertrophy and autophagy in the face of mechanical stress. However, whether the effect of HDL depends on Ang II or on gene regulation remains unclear and should be further investigated in future.

Our study revealed that the PI3K/Akt signalling pathway is involved in HDL-offered beneficial cardiac responses. This is supported by the cardioprotective properties of HDL. For example, HDL-stimulated activation of endothelial nitric oxide synthase is believed to be mediated through a PI3K/Akt-dependent mechanism 48. Activation of PI3K/Akt by HDL suppresses endothelial cell apoptosis 49, and HDL applied directly to isolated mouse cardiomyocytes promotes cell survival during hypoxia-reoxygenation through stimulating PI3K-Akt signalling cascade 14. Furthermore, it has been reported that treatment of H9c2 cardiomyocytes with IL-18 activated the PI3K-PDK-Akt/PKB signalling pathways that led to cardiac hypertrophy 50,51. It also has been known that the PI3K/Akt/mTOR pathway possesses a decisive role in the negative regulation of autophagy 25. However, it is still unknown whether HDL inhibited mechanical stress-triggered autophagy through Akt signalling pathway. Our further investigation revealed that HDL promoted Akt phosphorylation. The inhibition of Akt phosphorylation by MK2206 prevented HDL-elicited response against mechanical stress-induced autophage. These results suggested that HDL ameliorated autophagy *via* a PI3K/Akt-dependent mechanism. In this study, HDL treatment induced down-regulation of AT1 receptors and up-regulation of Akt phosphorylation. Also, Inhibition of Akt phosphorylation did not affect downregulation effect of HDL on the expression of AT1 receptors. It has been previously reported that inhibition of AT1 receptor attenuated mechanical stress-induced Akt phosphorylation. We therefore supposed here that HDL can reverse mechanical stress-induced cardiac hypertrophy and autophagy, at least in part, through inhibition of AT1 receptor-dependent Akt phosphorylation. The issue should be addressed in our future study.

In summary, our current study demonstrated, for the first time, direct beneficial effects of HDL against mechanical stress-induced autophagy in cardiomyocytes. Our results further demonstrated that HDL ameliorated cardiac hypertrophy and autophagy *via* an AT1 receptor- PI3K/Akt dependent mechanism. These findings indicate that treatment with HDL may help to prevent mechanical stress-induced cardiac hypertrophy and autophagy in the heart. Further study is warranted to elucidate the precise mechanism through which HDL suppresses mechanical stress-induced autophagy to maintain cardiac homeostasis under pressure overload.
